# Genome Sequence of a Heavy Metal-Detoxifying Actinomycete, Microbacterium proteolyticum ustc

**DOI:** 10.1128/mra.00349-23

**Published:** 2023-07-03

**Authors:** Qi-Zhong Wu, Wei-Qiang Lin, Wen-Zheng Du, Jian-Yu Wu, Wen-Wei Li

**Affiliations:** a School of Life Sciences, University of Science and Technology of China, Hefei, China; b CAS Key Laboratory of Urban Pollutant Conversion, Department of Environmental Science and Engineering, University of Science and Technology of China, Hefei, China; c Suzhou Institute for Advanced Research, University of Science and Technology of China, Suzhou, China; Indiana University, Bloomington

## Abstract

A complete genome is presented for Microbacterium proteolyticum ustc, a member of the Gram-positive order *Micrococcales* of the phylum *Actinomycetota* that is resistant to high concentrations of heavy metals and participates in metal detoxification. The genome consists of one plasmid and one chromosome.

## ANNOUNCEMENT

*Microbacterium* spp. include >110 cultivable strains isolated from anaerobic waste digesters (1), terrestrial ecosystems, food and clinical samples (2), and the microbiome of the gut in insects (3, 4), exerting profound environmental, ecological, and health impacts. It has been reported that *Microbacterium* strains can tolerate high concentrations of heavy metals and antibiotics and participate in the detoxification of heavy metals (5). However, the lack of a completely sequenced genome for this organism has been a limiting factor for the identification and modification of the key proteins involved in detoxification processes.

**FIG 1:**
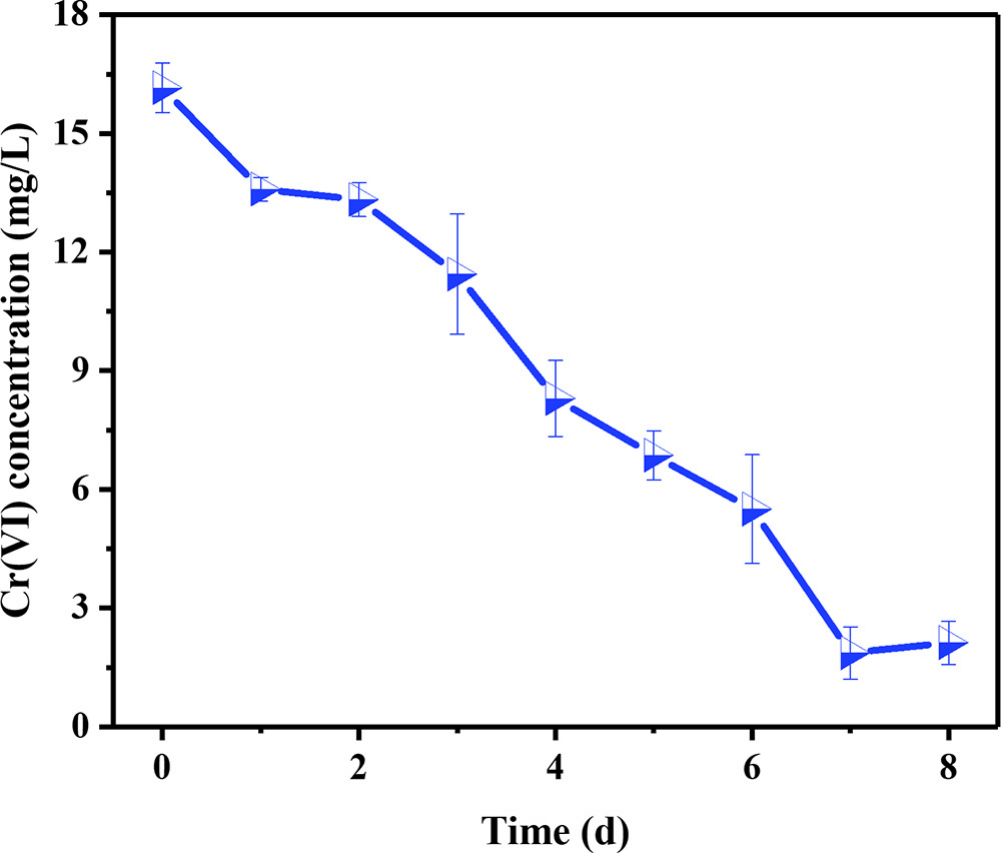
Reduction of hexavalent chromium by strain ustc. Samples were prepared in triplicate.

The strain ustc, which was isolated from pond soil on the campus of the University of Science and Technology of China (USTC) (Hefei, Anhui, China), was enriched by adding heavy metal chromium ions (1 mM Cr_2_O_7_^2−^) (6) to the lysogeny broth (LB) (7) medium for 24 h at 30°C. The ustc strain was identified as Microbacterium proteolyticum through 16S rRNA gene analysis after amplification. Cr(VI) (16 mg/L) was added to the serum vials containing 50 mL mineral medium (8) with 20 mM d-glucose. The strain was incubated in the mineral medium with an initial optical density at 600 nm (OD_600_) of 0.3. Samples were taken periodically, and the absorbance of supernatants at 540 nm was measured to monitor Cr(VI) levels (9). After 1 week, nearly all Cr(VI) had been reduced to the less toxic Cr(III) (Fig. 1).

Genomic DNA was extracted and purified using the Genomic-tip 20/G kit (Qiagen, Hilden, Germany) following the guidelines provided by the manufacturer. The sequencing libraries were generated using a rapid barcoding kit (SQK-LSK109) and sequenced in an R9.4 flow cell with an Oxford Nanopore Technologies system (10). The raw data from Nanopore sequencing were used by Guppy v3.2.6 software for base calling. In total, 162,675 reads (*N*_50_, 18,951 bp; *N*_90_, 2,717 bp) were acquired. AfterQC filters out bad reads (mean quality scores of <8 and lengths of <2,000 bp), detects and eliminates the sequencer’s bubble effects, trims reads at the front and tail, detects sequencing errors and corrects some of them, and finally outputs clean data; it generated 102,534 reads (*N*_50_, 20,252 bp; *N*_90_, 3,901 bp), which ensured that filtered reads could be assembled using Canu v1.5 (11).

Racon v3.4.3 software (12) was utilized to rectify the assembly outcomes, using three generations of subreads. For circularization and fine-tuning of the initiation site, Circlator v1.5.5 software was employed (13). Our work revealed two contigs, i.e., a chromosome measuring 3,834,223 bp and a plasmid measuring 136,212 bp. The genome's GC content is remarkably high, reaching 70.3% (Table 1).

**TABLE 1:** Assembly and annotation summary

Parameter	Finding for:		
	Whole genome	Chromosome	Plasmid
GenBank accession no.		CP121274	CP121273
Size (bp)	3,970,435	3,834,223	136,212
GC content (%)	70.31	71.10	65.26
No. of genes	3,785	3,628	157

Gene prediction was performed by using Prodigal v2.63 software (14), whereas rRNAs were predicted by Infernal v1.1.3 (15). The gene product function was predicted by employing DIAMOND v2.0.7 (16) (with an E value threshold of 1e−5), with the NCBI nonredundant protein, Swiss-Prot, Clusters of Orthologous Groups (COG), Kyoto Encyclopedia of Genes and Genomes (KEGG), and Gene Ontology (GO) databases. The match with the highest score was designated the final annotation of a specific gene in DIAMOND.

### Data availability.

The genome sequence has been submitted to GenBank under BioSample accession number SAMN34004116, BioProject accession number PRJNA950593, and SRA accession number SRR24745629, and the assembly is available under GenBank assembly accession number GCA_029639405.1.
